# A functional intervention within a cognitive approach to chronic cervical radiculopathy: Description of the non-surgical treatment arm in a randomised controlled trial evaluating the effectiveness of surgery

**DOI:** 10.1186/s12891-024-07743-0

**Published:** 2024-08-07

**Authors:** Kaia B. Engebretsen, Mirad Taso, Siri Bjorland, Hanne K. Jenssen, Helene Engberg Skaara, Jens Ivar Brox

**Affiliations:** 1 Department of Physical Medicine and rehabilitation, Oslo University Hospital, Oslo, Norway; 2 Faculty of Medicine, Institute for Clinical Medicine, University of Oslo, Oslo, Norway

**Keywords:** Cervical radiculopathy, Non-surgical treatment, Functional intervention, Cognitive approach, Graded activities, Individualised goals

## Abstract

**Background:**

Most patients with cervical radiculopathy improve within the first months without treatment or with non-surgical treatment. A systematic review concluded that these patients improve, regardless of their intervention. Still, many patients are offered surgery, despite limited evidence regarding the indications for surgical treatments. The aim of this article is to describe the intervention that is going to be followed in the non-surgical treatment arm of a randomised controlled trial (RCT) comparing the effectiveness of surgical and non-surgical treatment for patients with cervical radiculopathy.

**Methods:**

The non-surgical intervention is a functional intervention within a cognitive approach founded on previous experiences, and current recommendations for best practice care of musculoskeletal pain and cervical radiculopathy. It is based on the biopsychosocial rather than a biomedical perspective, comprises an interdisciplinary approach (physicians, physiotherapy specialists), and includes brief intervention and graded activities. The intervention consists of 6 sessions over 12 weeks. The primary goals are first, to validate the patients´ symptoms and build a therapeutic alliance, second, to explore the understanding and promote alternatives, and third, to explore problems and opportunities based on patients´ symptoms and function. Motivational factors toward self-management are challenging. We will attempt shared decision-making in planning progress for the individual patient and emphasise learning of practical self-help strategies and encouragement to stay active (reinforcing the positive natural course). General physical activities such as walking will be promoted along with simple functional exercises for the neck- and shoulder region. We will also explore social activity, comorbidities, pain location, sleep, and work-related factors. The health providers will set individualised goals together with each patient.

**Discussion:**

The aim of the intervention is to describe a functional intervention within a cognitive approach for patients with chronic cervical radiculopathy. The effectiveness of the present program will be compared to surgery in a randomised controlled trial.

**Supplementary Information:**

The online version contains supplementary material available at 10.1186/s12891-024-07743-0.

## Introduction

Cervical radiculopathy is usually caused by disc herniation, spondylosis, or a combination of herniation and spondylosis [1]. The underlying pathology is usually degenerative [1]. Cervical radiculopathy is defined as pain radiating to the arm in a strict nerve root pattern and with concomitant magnetic resonance imaging (MRI) findings [1, 2]. In addition, patients have neurological signs such as sensory disturbance, weakness and/or reduced tendon reflexes. Similar to patients with chronic neck pain without radiculopathy, these patients may also present with unspecific symptoms such as headache, dizziness and emotional distress. Functional limitations and disability are common with a peak in the middle age [3]. The prevalence was reported at 0.7 per 100 000 in a study from Rochester, Minnesota, USA applying strict diagnostic criteria [1]. Later studies based on less strict diagnostic criteria have reported a higher prevalence [4]. Age, gender and occupation are considered risk factors for radiculopathy caused by cervical spondylosis [5]. Factors reported to be associated with the development of cervical radiculopathy include prior lumbar radiculopathy, heavy lifting, operating heavy vibrating machinery and smoking [6]. The incidence of trauma preceding the onset of cervical radiculopathy is low [1, 6].

While a true natural course is difficult to outline, one systematic review concluded that patients with cervical radiculopathy improve over time regardless of their intervention assignment [7]. Most improvement occurs in the first 4–6 months and about 80% of patients are reported to recover completely within 2–3 years [8–10]. Variables reported to predict persistence of the condition include an absence of paresthesia, higher neck pain intensity, higher disability scores, reduced active rotation to the affected side and a workers´ compensation claim at baseline [8, 9]. More knowledge related to prognostic factors such as poor health, psychosocial factors and prior pain episodes are needed in order to guide clinical practice [6, 11].

The majority of patients with cervical radiculopathy are treated non-surgically [4]. A clinical trial from the Netherlands including patients with acute radiculopathy, found that symptoms are faster relieved by using a neck collar or by physiotherapy [10]. A clear consensus has not been reached regarding indications for surgery in this patient population [6], but patients with symptoms and signs of radiculopathy lasting 3 to 4 months are commonly referred for surgery. All patients are not improved by surgery. In 2022 there were about 1200 operations for cervical radiculopathy in Norway, slightly more associated with cervical spondylosis than disc herniation [12]. Although 78% were satisfied, about 40% did either not have a clinically relevant improvement, were unchanged or worse. The majority were operated at neurosurgical departments at public University Hospitals.

Systematic reviews suggest that non-surgical treatment strategies, such as medication, information related to the pain condition and clinical course, physical therapy including patient education, recommendation to stay physically active, exercises, manual therapy alone or in combination with different types of supervised exercises or the use of a cervical collar, may reduce neck pain and disability, improve function and quality of life [7, 10, 13–16]. An update of a previous systematic review proposed that muscle strength and endurance exercises for the cervico-thoracic spine and shoulders might be beneficial [16]. A review concluded that there is low level evidence that a multimodal intervention consisting of spinal and neurodynamic mobilisations and specific exercises are effective to reduce pain in patients with cervical radiculopathy [17].

The Danish clinical practice guideline recommends patient education, advice to stay physically active, different types of supervised exercises (SE) and manual therapy alone or in combination with exercises [18]. This guideline was established using the Grading of Recommendation Assessment, Development and Evaluation (GRADE) approach, and adheres to good clinical practice for patients with cervical radiculopathy. Despite previous studies and recommendations, high-quality evidence of the effectiveness of non-surgical treatments is lacking [19]. Recent systematic reviews are inconclusive and reinforce the knowledge of uncertainty of the benefit from multimodal conservative treatment including manual therapy [11, 20].

Interestingly, psychosocial factors have not been focused on in systematic reviews and recommendations, but Dedering et al. [21] found in a randomised study that neck-specific exercise training and prescribed physical activity that included additional a cognitive behavioral approach, decreased pain intensity. This is in agreement with studies suggesting that both exercises and psychosocial interventions may improve recovery for patients with chronic musculoskeletal pain (more than 3 months) [22, 23]. By example, a cognitive behavioral approach was equally effective compared with lumbar fusion in previous studies [24, 25], and more effective than usual care in patients with low back pain [26, 27]. Factors considered important are a good therapeutic alliance, focus on patient education, expectations including hope for change and recommendations to stay physically active [28, 29].

The main aim of this article is to describe the non-surgical treatment arm consisting of a functional intervention within a cognitive approach in one group of a randomised controlled trial (RCT) comparing the effectiveness of surgical and non-surgical treatment for chronic cervical radiculopathy.

## Patients and setting

In the RCT, patients with disabling radicular arm pain and magnetic resonance imaging (MRI) proving cervical disc herniation or spondylosis are randomised to receive either surgical or non-surgical treatment [30]. Patients referred to specialist healthcare at the Department of Physical Medicine and Rehabilitation and the Department of Neurosurgery, Oslo University Hospital for treatment for cervical radiculopathy at levels C5/C6 or C6/C7 are screened in order to include 200 patients [30]. The time from randomisation to treatment is between 2 and 3 weeks. The patients have follow-up assessments at 12, 26 and 52 weeks. All patients are informed that the outcomes of both treatments are considered to be good and that the researchers do not know if one treatment is more effective in reducing pain and improving function.

### Patient flow

Patients randomised to the non-surgical treatment group first have a consultation with a specialist in physical medicine and rehabilitation (SB or JIB). During the following 2 weeks, the patients then have two treatment sessions with a physiotherapist (HKJ or KBE) followed by another consultation with the physician after approximately 6 weeks. After 8 weeks, the patients meet the physiotherapist once more before a final consultation with the physician at 12 weeks. The physician and the physiotherapist usually discuss the patients´ progress.

### Content of the functional intervention

A functional intervention within a cognitive approach is a patient-centred approach which focus on validation of the patient´s symptoms, building a therapeutic alliance, addressing fear avoidance and activity, and prescribing general activities and some simple exercises [26, 31]. The intervention in the present study is based on a biopsychosocial approach addressing both physical and cognitive factors within an expanded outline of the framework of brief intervention [32, 33]. It is founded on previous experience, studies on cervical radiculopathy and neck pain, and current recommendations for best practice care for musculoskeletal pain [23, 30, 34]. The present interdisciplinary intervention comprises experienced physicians and physiotherapists and consists of six preplanned sessions of up to 1 h each.

## Validation of patients’ symptoms and therapeutic alliance

Understanding the patient’s perspective and providing an empathetic attitude are essential components of building a therapeutic alliance. Therefore, the first session with the physician starts with a thorough clinical examination and explanation of the MRI findings and reported symptoms. An important goal of the first consultation, is to achieve a therapeutic alliance [29]. The natural course of cervical radiculopathy and the content of the program are explained. Patients present their history related to their current condition and their expectations for the future. They are encouraged to describe their previous treatment experiences and express their beliefs and hopes for change of symptoms. The physician attempts to understand patients´ reflections related to their pain, function and worries about the future. Patients’ understanding of their symptoms are individually explored to enhance motivation to change and help to identify and reduce possible barriers to the natural course [35, 36]. We try to modify the patients understanding of the MRI findings. By example degeneration with foraminal narrowing are often observed also at the contralateral side or at the level above without concomitant symptoms and signs, questioning the strict association of imaging and symptoms and signs.

**Fig. 1:**
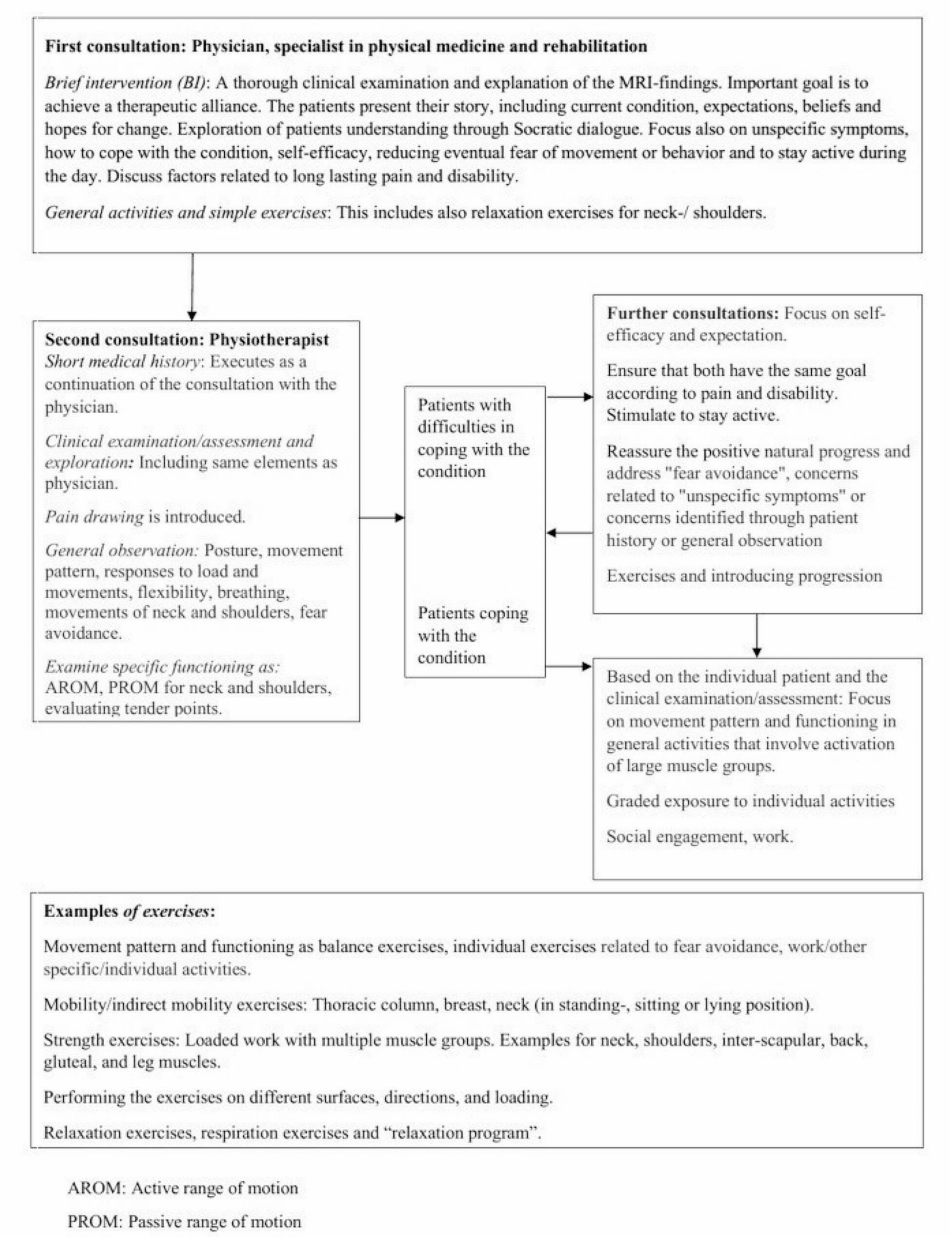
Functional intervention for patients with chronic cervical radiculopathy

We use a Socratic dialogue with open questions which facilitates critical thinking in order to promote an alternative understanding. The Socratic dialogue assists to guide the patient towards solutions of their health-issues and unspecific symptoms [37]. The patients´ problems are explored and opportunities such as practical self-help strategies reinforcing a positive natural course, are emphasised [32, 38]. In collaboration with the individual patient, we identify and help to interpret factors perceived as activity and participation barriers [39, 40]. These steps are not always straightforward, as illustrated in Fig. 1.

### Avoidance and activity

Before entering the study, patients may have been recommended restrictions in physical activity such as being cautious, protecting their neck, and staying away from ordinary physical activity.

Pain location, including radiating pain, and active and passive range of neck motion, are considered. A pain drawing is used to assess if regional- or multisite pain accompany the radicular pain [41]. A recent study showed that in patients with cervical radiculopathy, more widespread pain was associated with more headache, neck- and arm pain and disability [42]. Emotional distress is associated with more widespread pain [43].

The physiotherapists observe the patients performing some general functional movements, including jumping, limping, picking up a weight from the floor and carrying objects. The rationale for performing these functional movements is to examine if symptoms are reproduced and if fear avoidance behavior appears.

We promote graded exposure to activities that are avoided [44, 45]. Avoidance is a common response to pain and may contribute to disability [45].

### Exercises

Exercises are provided in order to improve function and reduce painful muscle activity [46]. Exercises include simple shoulder roll, scapular retraction and relaxation exercises to improve motor control and reduce co-activity of supporting muscles [46]. The rationale for these exercises is to improve cervical muscle balance and to achieve relaxed postures before movement- and functional training [3, 26]. Dynamic exercises are introduced to activate and stretch the neck and shoulder muscles and some components of postural ergonomics based on the patient´s general functional status [47]. Scapular strengthening and deep flexor exercises have also provided some benefit [48]. The exercises are individually tailored based on patient’s response [21]. Tender or trigger points in the muscles may contribute to pain and particularly upper trapezius muscle activity are related to neck pain, headaches, and emotional distress [49, 50]. Manual treating of these points, may improve local pain in the short to medium term [51].

The patients will receive written instructions including pictures illustrating the exercises.

Additionally, patients are recommended to try to carry out general physical activities daily.

Patience and adherence to exercise- and activities have shown to improve clinical outcomes [37]. Individual adjustments are made during the treatment period to help the patient feel validated and taken care of in the therapeutic setting [52].

#### A case description to illustrate the proposed intervention

The case description is not a patient who is going to be included in the trial, but could have been according to the inclusion criteria.

Ann is a 52 year old woman, working with children aged 3 to 6 years in a kindergarten. She experienced neck pain for the first time 1 year ago, but the pain became more severe over the 6 months and was accompanied by radiating pain in the left arm. The x-ray of the neck showed general degeneration indicative of normal age related changes. MRI showed a disc herniation at level C6/C7 affecting the left C7 nerve root. Her pain radiated into the shoulder and down the arm all the way to 3.- 5.finger with accompanying numbness. She also experienced increased local neck pain and stiffness when trying to move her head and reported aching in the muscles in the neck- and shoulder region on the left side. This included some trigger points in the infraspinatus muscle. Her sleep was disturbed and she was constantly tired, waking up with a headache almost every morning. She had been on sick-leave for the last 3 months.

Ann had previously been to a physiotherapist two times a week and performed some exercises over the last 5 months. Her family physician and the physiotherapist told her to be careful with her neck and to avoid activities such as hiking, riding, swimming and bicycling. She followed their recommendations, but had been out for a walk now and then. She was afraid of doing something “wrong” for her neck. Her pain condition was not improved, and she was afraid of never getting better. This made her feel depressed.

When she spoke about her family (husband and two children, 15 and 17 years old), she expressed worries about her 15 year old son, who was diagnosed with diabetes 2 years ago. She was emotionally distressed almost all the time. Drugs only provided short-term pain relief. She previously saw some friends now and then, but was seldom social over the last 2 months. She reported severe neck pain, strong fear avoidance beliefs for physical activity, severe emotional distress and severe neck disability including headache and dizziness. She had low expectations for non-surgical treatment.

#### The approach for Ann

During the first session, the physician listened to Ann´s story, explored her understanding of the condition in a Socratic dialogue, and performed a thorough clinical examination. The physician aimed to get a good therapeutic alliance and to identify her concerns, thoughts and beliefs regarding her pain condition. Her expectations regarding non-surgical treatment and her MRI findings were discussed. Ann became more motivated and willing to try it. She thought that the non-surgical treatment and exercises would be good for her. However, she had already been to a physiotherapist and experienced increased pain and numbness related to these visits. Her beliefs, which were largely formed from a biomechanical perspective and based on the MRI findings, made her think that surgery may be the best choice. Ann´s previous experiences and expectations related to her pain condition, dominated her beliefs. The physician thus spent the first session reassuring her that being active and moving her head was safe, and avoidance was not recommended. During the first session, the physician also asked about her worries for her son and explained how her worries and anxiety might increase pain and inhibit the natural course. The physician attempted to find more positive alternative thoughts that she could believe in.

Ann had 3 sessions together with the physiotherapist. The physiotherapist continued the treatment with a short clinical examination, listened to her worries, discussed her pain condition and tried to deal with it in an optimistic manner. Active range of motion and functional movements were registered. Ann moved her head carefully. She had a maladaptive movement pattern with elevated shoulders, especially the left one. The increased tension in neck muscles and her head in a forward posture, led to muscular imbalances, which also affected her breathing. We discussed her body responses in front of a mirror. She was motivated for some simple exercises and tried 3 relaxation exercises as shoulder roll, shoulder blade contraction in standing position and arm swinging (additional file 1). In collaboration, we decided that 3 sets of 5 repetitions at least twice a day, could be a good start. Of importance was also to improve the diaphragm breathing. Additionally, she started to go out for a walk, swinging with her arms as naturally as possible while walking. She began with 15 min a day and could proceed to 15 min twice a day.

At the next physiotherapy session she had increased the daily walking distance and had less pain. She also felt that the relaxation exercises was good for her increased muscle tension. Ann was motivated for more exercises and instructed in 2 dynamic stretching exercises for the neck muscles (upper trapezius and levator scapula) and 2 exercises for the interscapular muscles (total 4 exercises, 3 sets, 8–10 repetitions) (additional file 1). This was homework and goal for the next session along with increased general physical activity. She also wanted to try her bicycle. Of importance was that she gained confidence in being more active. At the 3.session with the physiotherapist (last), she had less pain, her sleep had improved and her headache reduced. She wanted some strengthen exercises and was introduced to low row with a thin elastic rubber band and overhead arm press with light weight (0.5-1.0 kilo) (3 sets, 8–10 repetitions) (additional file 1). She could step by step increase the weight. After this session also numbness and fear of movements had decreased.

Gradually, she experienced less pain when she performed activities. Some of her negative thoughts regarding the future did not occur or become a problem, and her son with diabetes was doing better. Ann was more active and in a better mood. In addition to be more physically active, she met some friends again, and gradually planned to return to work. After the 6. session (12 weeks), she had some pain now and then, but her attitude to the future was more optimistic. She still worried about her son, but her worries did not dominate her daily life in the same way they did previously.

The physician and physiotherapist set individualised goals together with Ann. When she experienced self-efficacy and realised that it was safe to be active, her movements changed. She felt better, and her sleep improved. Her fear avoidance beliefs decreased, which was important to her improvement. Thoughts and behaviors changed, pain and disability was reduced and emotional distress gradually relieved.

## Discussion

The main aim of this article is to describe the content and the conduction of a functional intervention within a cognitive approach in a randomised controlled trial comparing non-surgical and surgical treatment for chronic cervical radiculopathy [30]. The patients represent a selected group, as all are candidates for surgery [53]. The effectiveness of this treatment compared to other non-surgical treatments or the natural course, has not been evaluated.

The intervention is briefly described in line with the study protocol [30]. Because all involved in the treatment are educated in cognitive behavioral therapy, it is apparent that elements from this approach are applied. Our approach is comparable to the classification-based cognitive functional therapy in the study by Fersum et al. [26], in patients with chronic low back pain. Both interventions focus on targeting important factors for change. We apply a Socratic dialogue to promote an alternative understanding of unspecific symptoms similar to the comprehensive interview and reflective communication in the low back pain study. Because the patients´ main problem are considered to be cervical radiculopathy and because they are referred for surgery, the Socratic dialogue is demanding.

The non-surgical intervention described, is not based on the MRI findings, but considered for each patient in accordance with their individual pain and disability [30, 54]. It is common to presume that verifying the patient group by MRI provides a relatively homogenous group of study participants, but patients can present with substantially different pain and disability levels despite having similar MRIs.

Patient expectations as described by Taso et al. [53] are considered as an important prognostic factor. Previous research has shown that the best predictor of functional outcomes with a non-surgical approach is pre-treatment patient expectations [55]. Long-lasting pain and previous experiences with health providers are negative prognostic factors and often increase patient worry. Many patients need individualised reassurance to experience hope and improve self-efficacy. Maxwell et al. [56] found that for shoulder pain, openness to other treatment options than operative management was often influenced by factors such as understanding of pain, prior experiences and treatment expectations. A long natural course may hamper motivation for non-operative treatment and increase motivation for surgery.

We use a broad assessment at baseline to guide in shared decision-making. This is helpful in order to develop an individual treatment plan. Functional exercises and graded exposure are used rather than traditional exercise principles [23]. It is debated whether specific exercises exist for patients with cervical radiculopathy, but it is suggested that functional exercises for the shoulder and neck region may be helpful [21].

The brief intervention consists of few sessions and positive results are reported in patients with low back pain, but has not been evaluated in patients with cervical radiculopathy [33]. Patients with cervical radiculopathy have a different pathology, but muscular dysfunction, fear avoidance beliefs and other factors that accompany persistent pain, are similar. Research on the brief intervention approach in patients with low back pain, showed that it elicited higher return-to-work rates compared to treatment as usual [39].

### Strengths and limitations

In RCTs, the management strategies are often reported in terms of the key principles applied and not individualised to each patient. We have described a pragmatic functional intervention within a cognitive approach that addresses the individual patient based on the framework displayed in Fig. 1. The intervention may be reproducible. The effectiveness of this intervention are compared with surgery [30]. We have included a description of a case to illustrate a patient course. Patients with a favorable outcome may have had a positive natural course, perceived a good alliance with their health providers or experienced hope for recovery. These factors are considered to be important components of the placebo response. The specific outcomes of the functional intervention are challenging to discriminate from placebo. It is difficult to find a valid placebo intervention for comparison. Future pragmatic randomised controlled trials should compare the effectiveness of the functional intervention with usual care, mobilisation or muscular strengthening. In studies of patients with low back pain, similar interventions have reduced pain-related disability and fear avoidance [24, 57–59].

Qualitative studies may help clinicians to develop better non-surgical interventions [60].

### Clinical implications

The described functional intervention within a cognitive approach consists of rather few sessions and its effectiveness is compared with surgery in randomised controlled trial.

### Electronic supplementary material

Below is the link to the electronic supplementary material.

Additional file 1

## Data Availability

The dataset analysed during the current study are not publicly available due to the unpublished randomised controlled trial comparing the effectiveness of surgical and nonsurgical treatment for cervical radiculopathy by Mirad Taso et al., which this study is related to, but are available from the corresponding author on reasonable request.
