# Supplementary material for: A functional intervention within a cognitive approach to chronic cervical radiculopathy: Description of the non-surgical treatment arm in a randomised controlled trial evaluating the effectiveness of surgery
Source: BMC Musculoskelet Disord. 2024 Aug 7;25:629. doi: 10.1186/s12891-024-07743-0 (PMC11308144; doi:10.1186/s12891-024-07743-0)
Supplement: Supplementary file 1 — Additional file 1 [file 12891_2024_7743_MOESM1_ESM.docx]

Additional file 1
Table 1 Exercises for Ann

| **Exercise** | **How** | **Reps** | **Sets** |
| --- | --- | --- | --- |
| Shoulderblade contraction | Stand upright with your arms down by your sides. Bring your shoulder blades together and slightly down. | 5 | 3 |
| Shoulder roll | Roll both shoulders simultaneously. Aim for large, circular movements. Roll in one direction, then switch to the opposite direction. Keep your arms hanging by your sides throughout the exercise. | 5 | 3 |
| Standing arm swing | Stand with the arms hanging straight down along your side. Relax the shoulders and swing the arms back and forth. |  | Daily, and when out walking |
| Stretching the neck  (m upper trapezius) | Stand or sit. Hold one hand on collar bone. Simultaneously bend and rotate your head in the opposite direction to the position of your hand. Gaze down and feel a stretch on the front of your neck. Hold for 20-30 seconds. | 5 | 1 |
| Stretching the neck  (m levator scapula) | Hold your hands behind your back. Lower your shoulders and rotate your head towards one side first until you feel tension in the back of the neck and then the other side. Hold the position for 20-30 seconds and take a few deep breaths. Repeat on the opposite side. | 5 | 1 |
| Scapula retraction | In prone postion, place your arms down along your body, your forehead rests on a towel Keep your arms to the ground while you lift your shoulders and pull the scapula medially and downwards. Hold this tension a few seconds and then repeat. | 8 | 3 |
| Diagonal lift, standing on all fours | Start on all fours with your face facing the floor. Stretch one arm and the opposite leg to extend of your body. Repeat the movement with opposite arm and leg. | 8 | 3 |
| Standing low rowing w/thin elastic band | Attach a thin elastic band to the door. Stand with your feet apart, facing with a handle in each hand. Hold your arms straight in front of you and pull the handles toward your abdomen. Slowly return to the start position and repeat. | 8 | 3 |
| Shoulder press | Stand upright. Hold a 0.5-1.0 kilo weight with one hand, the palm facing forwards. Press the weight straight upwards. Slowly return to the starting position and repeat on the opposite side. | 8 | 3 |
